# Skeletal Muscle Density as a Predictor of Prognosis and Physical Reserve in Patients with Cancer of Unknown Primary

**DOI:** 10.3390/jcm14092947

**Published:** 2025-04-24

**Authors:** Kwonjae Lee, Se Jun Park, Joori Kim, Sook Hee Hong, In-Ho Kim, Jieun Lee, Myung Ah Lee, Kabsoo Shin, Han Song Mun

**Affiliations:** 1 Division of Medical Oncology, Department of Internal Medicine, Seoul St. Mary’s Hospital, College of Medicine, The Catholic University of Korea, Seoul 06591, Republic of Korea; kwonjae0416@naver.com (K.L.);; 2 Cancer Research Institute, College of Medicine, The Catholic University of Korea, Seoul 06591, Republic of Korea; 3 Department of Radiology, College of Medicine, Seoul St. Mary’s Hospital, The Catholic University of Korea, Seoul 06591, Republic of Korea

**Keywords:** cancer of unknown primary, skeletal muscle density, overall survival

## Abstract

**Introduction:** The Eastern Cooperative Oncology Group (ECOG) Performance Status (PS) is widely used to assess patient status but relies on subjective judgment and may not fully reflect their physical reserve. While studies have shown that skeletal muscle quality and quantity are associated with patient prognosis, their role in cancers of unknown primary (CUP) remains unclear. Therefore, this study aimed to investigate whether computed tomography (CT)-based skeletal muscle indicators reflect physical reserve and their prognostic value in patients with CUP. **Methods:** This study enrolled 184 patients with CUP, comprising both inpatients and outpatients, who were diagnosed at Seoul St. Mary’s Hospital between 1 January 2008, and 30 June 2024. Overall survival (OS) was evaluated using the Kaplan–Meier method and analyzed using the log-rank test. Univariate and multivariate analyses were performed using Cox proportional hazard models. Statistical significance was defined as *p* < 0.05. Correlation analyses were conducted to evaluate the relationships between skeletal muscle density (SMD), skeletal muscle index (SMI), and other prognostic factors. **Results:** SMD was positively correlated with SMI and negatively correlated with age, neutrophil-to-lymphocyte ratio, Charlson Comorbidity Index (CCI), and ECOG-PS. Jonckheere’s trend test revealed that SMD decreased significantly as CCI and ECOG-PS increased (*p* < 0.001), indicating that a higher comorbidity burden and poorer performance status were associated with lower SMD. Both ECOG-PS and SMD were identified as prognostic factors in the univariate analysis of survival; however, only SMD demonstrated statistical significance regarding prognostic value in the multivariate analysis (*p* = 0.004) **Conclusions:** SMD, as a measure of muscle quality, demonstrates superior prognostic value compared to the subjective ECOG-PS and may serve as a reliable objective tool for assessing physical reserve in patients with CUP.

## 1. Introduction

Cancer of Unknown Primary origin (CUP) is defined as a biopsy-proven malignancy without an identified primary site of origin despite comprehensive evaluation, and it accounts for approximately 2–5% of all cancers [1]. Although the incidence of CUP has decreased to 1–2% owing to advances in diagnostic techniques [2], its prognosis is generally poor.

Patients with CUP can be categorized into favorable and unfavorable prognostic groups. Within this heterogeneous disease entity, certain subsets exhibit distinct clinical and pathological features that enable a presumptive identification of the primary site, even if it remains undetected [3]. These favorable-risk subsets comprise approximately 10–20% of CUP cases. In contrast, patients who do not meet the criteria for favorable subsets are classified into the unfavorable prognostic group, which is generally associated with poorer clinical outcomes and a median survival ranging from approximately 1.7 to 7.2 months [4]. Patients in the favorable group typically experience better survival outcomes when treated with site-specific or tailored therapeutic strategies [1,4,5,6,7].

Due to the heterogeneous nature of CUP, universal clinical factors [8,9] such as the Eastern Cooperative Oncology Group-Performance status (ECOG-PS), age, lactate dehydrogenase (LDH), histology, disease extent, metastatic pattern [10,11], and neutrophil-to-lymphocyte ratio (NLR) [12] have been evaluated as prognostic indicators. A representative survival prediction model that uses these prognostic factors is the Culine model [13], along with other nomograms [14].

The ECOG-PS has been extensively utilized for decades as a rapid, straightforward, and globally recognized tool for assessing the performance status of patients with cancer. Despite its widespread acceptance, ECOG-PS possesses inherent limitations. Its simplified 0–5 categorical scale may inadequately reflect the nuanced clinical heterogeneity among patients and is prone to inter-observer variability due to its reliance on subjective clinical judgment. Furthermore, ECOS-PS does not adequately capture individual differences in physical reserve, functional capacity, or underlying comorbidities, even among patients assigned the same ECOG-PS score. Although alternative approaches have been proposed to address these shortcomings, they have not yet achieved broad clinical integration [15].

Muscles not only enable movement but also influence multiple organs and systems throughout the body, including the bones, blood vessels, nerves, liver, heart, pancreas, colon, and brain. According to the European Working Group on Sarcopenia in Older People (EWGSOP), sarcopenia is defined as an accelerated loss of skeletal muscle mass and function associated with aging and immobility [15]. The rate of muscle loss can vary depending on activity level, comorbidities, nutrition, and other factors. The diagnosis of sarcopenia requires a multifactorial assessment that includes both quantitative measurements of muscle mass and evaluations of muscle function [15]. However, performing such assessments can be particularly challenging in patients with advanced cancer, who are often compromised both physically and psychologically.

However, in recent years, computed tomography (CT) imaging has been used to assess both the quality and quantity of skeletal muscles [16,17], with findings showing that these measurements are associated with the prognosis of patients with various cancers [18,19,20,21,22,23,24]. Muscle quantity is commonly assessed using the Skeletal Muscle Index (SMI), while muscle quality is evaluated through Skeletal Muscle Density (SMD).

Despite growing evidence in other cancer types, the prognostic significance of these skeletal muscle indicators in patients with CUP remains unclear. Furthermore, their relationship with other markers of physical reserve has yet to be clarified in this patient population.

Therefore, this study aimed to analyze the correlation between CT-based skeletal muscle indicators and other clinical factors, including ECOG-PS, in patients with CUP and to evaluate the prognostic significance of skeletal muscle indicators.

## 2. Materials and Methods

### 2.1. Study Design

This is a retrospective observational study conducted at a single center.

### 2.2. Patients Sample

This study analyzed 184 patients with CUP who were treated at Seoul St. Mary’s Hospital between 1 January 2008 and 30 June 2024. Both inpatient and outpatient cases were included.

### 2.3. Inclusion Criteria

Adults aged 20 years or older, regardless of sex, with histologically confirmed malignancies were included in the study. Eligible patients had undergone comprehensive diagnostic evaluations, including imaging studies, but were ultimately diagnosed with CUP. Additionally, patients were required to have available data on disease progression and survival status for inclusion in the analysis.

### 2.4. Exclusion Criteria

Individuals younger than 20 years of age; patients who were initially diagnosed and treated as having CUP but were subsequently found to have a confirmed primary tumor site; patients who were clinically and radiologically suspected of having CUP but lacked histological confirmation of malignancy; and patients for whom data on disease progression or survival status were unavailable were excluded.

### 2.5. Data Collection

All patients underwent a comprehensive diagnostic evaluation, including history taking, physical examination, basic laboratory tests, chest radiography, and CT scans of the chest, abdomen, and pelvis. The extent of disease at the time of diagnosis was also assessed. Treatment modalities included chemotherapy, radiotherapy, surgery, and concurrent chemoradiotherapy. In addition, data on patient demographics (age and sex), date of cancer diagnosis, date of death were collected.

To assess patients’ comorbidity burden and functional status, the Charlson Comorbidity Index (CCI) and ECOG-PS were evaluated based on a retrospective review of medical records. Documentation of past medical history and ECOG-PS assessments by the attending physicians at the time of diagnosis were used for this purpose.

#### 2.5.1. Charlson Comorbidity Index

The CCI was calculated as follows. Age was incorporated by adding 1 point for each decade over 50 years, up to a maximum of 4 points. The index includes the following comorbidities: myocardial infarction (1 point), congestive heart failure (1), peripheral vascular disease (1), cerebrovascular accident (1), dementia (1), chronic obstructive pulmonary disease (1), connective tissue disease (1), peptic ulcer disease (1), liver disease (mild: 1; moderate to severe: 3), diabetes mellitus (without complications: 1; with end-organ damage: 2), hemiplegia (2), moderate-to-severe chronic kidney disease (2), solid tumors (localized: 2; metastatic: 6), leukemia (2), lymphoma (2), and acquired immune deficiency syndrome (6). The total score ranges from 0 to 37, with higher scores indicating a greater comorbidity burden and increased risk of mortality [25].

#### 2.5.2. ECOG Performance Status

ECOG Performance Status was assessed according to the following criteria: 0 indicates the patient is fully active and able to carry on all pre-disease activities without restriction; 1 indicates some limitation in physically strenuous activity, though the patient is ambulatory and capable of performing light housework; 2 indicates the patient is ambulatory and capable of all self-care but unable to carry out any work-related activities, spending more than 50% of waking hours out of bed; 3 indicates the need for some assistance with self-care, with more than half of the day spent in bed or a chair; 4 indicates complete dependence, with the patient unable to perform any self-care and confined to bed or chair for the entire day; and 5 indicates death [26].

### 2.6. Skeletal Muscle Evaluation

Skeletal muscle radiodensity was assessed using abdominopelvic CT images obtained at the time of diagnosis. The cross-sectional areas of the rectus, transverse, and oblique abdominal muscles, psoas muscles, and paraspinal muscles were measured at the L3 vertebral level using a commercial deep learning-based software (https://claripi.com/ accessed on 13 March 2025, ClariMetabo, ClariPi, Seoul, Republic of Korea). A threshold of −29–150 Hounsfield units (HU) was applied to identify muscle areas. The L3 skeletal muscle area represents whole-body muscle mass [27]. The schematic diagram of the deep learning-based muscle analysis method is shown in Figure S1, and the analysis results are reported in the format of Figure S2. The SMD was assessed as the mean radiodensity (HU) of the entire cross-sectional muscle area at the L3 level. The skeletal muscle area was divided by the square of the patient’s height to normalize the L3 SMI and was calculated as follows: lumbar skeletal muscle cross-sectional area (cm^2^)/height (m^2^).

ClariMetabo utilizes two deep learning (DL) models to quantify abdominal fat and muscle components in CT images. The first model, trained on MIP CT images from over 800 patient scans, localizes the vertebral body (T12–L4) with a categorical accuracy of 99.1%. The second model, a 2D U-Net trained on more than 40,000 CT images, automatically segments abdominal fat and muscle, achieving a Dice similarity coefficient of 0.96–0.98. To ensure consistency in image analysis, a predefined HU threshold (−29 to 150 HU) was applied, and an experienced radiologist visually reviewed the segmentation results to verify accuracy.

### 2.7. Statistical Analysis

Overall survival (OS) was estimated using the Kaplan–Meier method and compared between groups using the log-rank test. OS was defined as the time from the date of cancer diagnosis to the date of death from any cause. Progression-free survival (PFS) was defined as the time from the initiation of chemotherapy to documented disease progression or death, whichever occurred first.

To explore the relationship between SMD and clinical variables, Pearson’s correlation analysis was used for continuous variables with normal distribution, while Spearman’s rank correlation was applied for ordinal or non-normally distributed variables. Jonckheere’s trend tests were performed to assess the trend in SMD across ordered levels of CCI and ECOG-PS.

Survival outcomes were analyzed using the Cox proportional hazards regression model. Variables with a *p*-value < 0.1 in univariate analysis were included in the multivariate model, and those with a *p*-value < 0.05 were considered statistically significant. Hazard ratios (HRs) and 95% confidence intervals (CIs) were reported for each variable.

All statistical analyses were performed using GraphPad Prism (version 8.4.2) for descriptive statistics and survival plots, and R software (version 4.4.1) with the ‘survival’ and ‘survminer’ packages for regression modeling and trend analysis. A two-sided *p*-value < 0.05 was considered statistically significant.

### 2.8. Study Approval

The present study was conducted following the official approval of the Institutional Review Board of the Catholic University of Seoul Saint Mary’s Hospital. Given that all data analyzed were previously recorded in patients’ medical records exclusively for routine clinical care, the requirement for informed consent was formally waived. Ethical clearance for this research was duly granted and registered under the approval code KC24RISI0356 on 4 June 2024.

## 3. Results

### 3.1. Baseline Characteristics

Patients diagnosed with CUP had a median age of 68 years (range 21–90), with 44.6% of the patients being female and 55.4% being male, respectively (Table 1). The most common histological findings were adenocarcinoma (31.5%), squamous cell carcinoma (19.0%), and poorly differentiated carcinoma (17.9%). Regarding the ECOG-PS, 157 patients (85.3%) had a score of ≤2, while 27 patients (14.7%) had a score of ≥3. The median CCI was 3, with 119 patients (64.7%) having a CCI ≤ 3 and 65 patients (35.3%) having a CCI > 3.

At diagnosis, 100 patients (54.3%) had an NLR < 5, and 84 patients (45.7%) had an NLR ≥ 5, with a median NLR of 4.78 (range, 0.6–35.3). A total of 79 patients (42.9%) had single or oligometastasis, while 105 patients (57.1%) had multiple metastases (Table S1). Additionally, 108 patients (58.7%) received some form of treatment, whereas 76 patients (41.3%) received only supportive care (Tables S2–S4).

CT-based SMD was measurable in a total of 132 patients, with a median SMD of 39. Among them, 63 patients (34.2%) had an SMD > 39, and 69 patients (37.5%) had an SMD ≤ 39. CT-based SMI was measurable in a total of 125 patients, with a median SMI of 43.3. Among them, 62 patients (33.7%) had an SMI > 43.3, and 63 patients (34.2%) had an SMI ≤ 43.3.

### 3.2. Correlation Analysis—Skeletal Muscle Indicators and Other Clinical Factors

**Figure 1:**
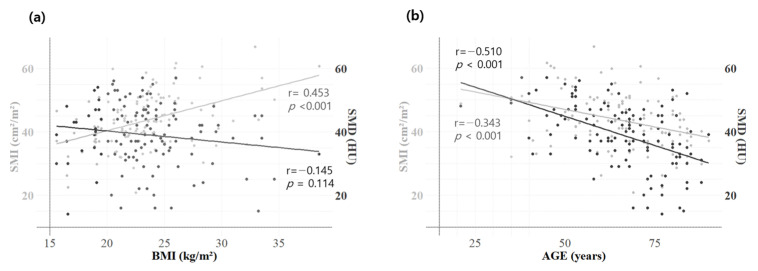
Correlation between BMI, SMI, and SMD of the study patients. Pearson correlation analysis of skeletal muscle indicators: The graphs illustrate the correlation between BMI and SMI/SMD (**a**) and between age and SMI/SMD (**b**). BMI exhibited a significant positive correlation with SMI (gray line, r = 0.453, *p* < 0.001) and a negative, though not significant, correlation with SMD (black line, r = −0.145, *p* = 0.114). Age showed a significant negative correlation with both SMI (r = −0.510, *p* < 0.001) and SMD (r = −0.343, *p* < 0.001). Body mass index, BMI; skeletal muscle index, SMI; skeletal muscle density, SMD.

Both SMD and SMI showed significant negative correlations with age and NLR, as determined by Pearson’s correlation coefficient (Table 2). BMI demonstrated a significant positive correlation with SMI (r = 0.453, *p* < 0.001) and a negative, though not significant, correlation with SMD (r = −0.145, *p* = 0.114) (Figure 1a). Age demonstrated a significant negative correlation with both SMI (r = −0.343, *p* < 0.001) and SMD (r = −0.510, *p* < 0.001) (Figure 1b).

**Figure 2:**
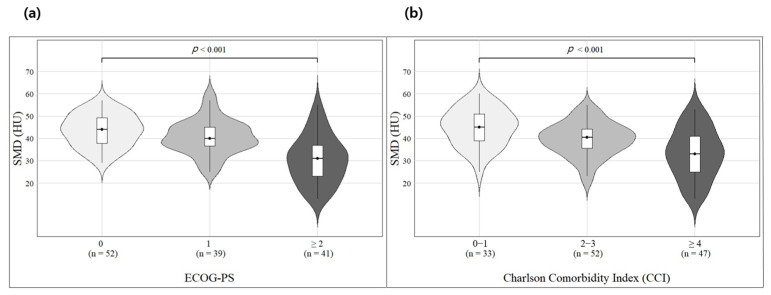
Jonckheere’s trend test between SMD, CCI, and ECOG-PS of the patients (**a**,**b**).

In Spearman correlation analysis, both SMD and SMI exhibited negative correlations with CCI and ECOG-PS, but the strength and significance of the correlation were higher for SMD (Table 2). Furthermore, Jonckheere’s trend test revealed that as CCI and ECOG-PS scores increased, SMD decreased significantly (*p* < 0.001), indicating that a higher number of comorbidities and poorer ECOG-PS scores were associated with lower SMD (Figure 2).

When correlations were examined within male and female subgroups, SMD consistently exhibited significant associations with both CCI and ECOG-PS in both sexes. In contrast, SMI showed significant correlations with CCI and ECOG-PS only in the male subgroup. These findings suggest that SMD reflects a patient’s functional status consistently, regardless of sex.

Jonckheere’s trend test indicates a relationship between SMD and CCI as well as ECOG-PS, showing that as the CCI and ECOG-PS increase, SMD tends to decrease. CCI and ECOG-PS were analyzed for 132 individuals with available SMD data; 33 were in the CCI 0–1 group, 52 in the CCI 2–3 group, and 47 in the CCI ≥ 4 group. ECOG-PS analysis showed that 52 individuals were in the ECOG-PS 0 group, 39 in the ECOG-PS 1 group, and 41 in the ECOG-PS ≥ 2 group. Skeletal muscle density, SMD, Charlson Comorbidity Index, CCI; Eastern Cooperative Oncology Group Performance Status, ECOG-PS.

### 3.3. Survival Analysis Based on SMD and Other Clinical Factors

**Figure 3:**
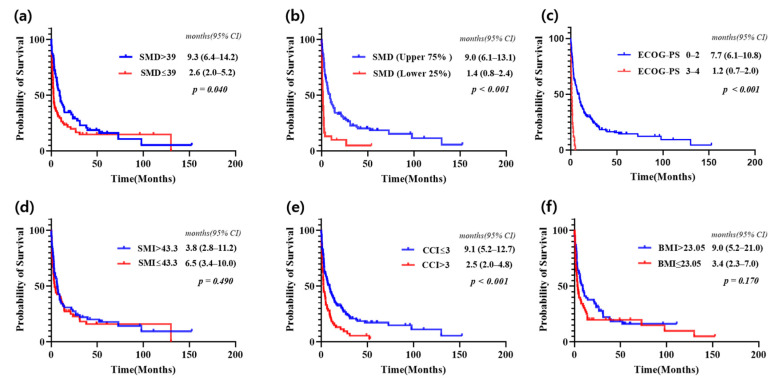
Kaplan–Meier curves showing overall survival for all patients with CUP in the study (**a**–**f**).

Survival was analyzed using the Kaplan–Meier method after dividing patients into two groups based on various clinical factors (Figure 3). When divided by the median value, patients in the SMD > 39 group had a significantly longer median overall survival (mOS) compared to the SMD ≤ 39 group (9.3 months [95% CI 6.4–14.2] vs. 2.6 months [95% CI 2.0–5.2], *p* = 0.04). Similarly, when patients were divided based on ECOG-PS into 0–2 and 3–4 groups, the 0–2 group had significantly longer mOS (7.7 months [95% CI 6.1–10.8] vs. 1.2 months [95% CI 0.7–2.0], *p* < 0.001). Patients with CCI ≤ 3 had a significantly longer survival compared to those with CCI > 3 (9.1 months [95% CI 5.2–12.7] vs. 2.5 months [95% CI 2.0–4.8], *p* < 0.001). However, when patients were divided by the median for SMI and BMI, no significant differences in survival were observed between the groups.

When SMD was divided into groups based on the upper 75% group (above the 25th percentile) and the lower 25% group (below the 25th percentile), the survival differences became more pronounced (Figure 3b). The mOS for the upper 75% group was 9.0 months (95% CI, 6.1–13.1), whereas the mOS for the lower 25% group was 1.4 months (95% CI, 0.8–2.4), with *p* < 0.001. These findings indicate that lower SMD values are strongly associated with significantly worse prognosis.

The median overall survival for all CUP was 5.0 months (95% CI 3.4–9.0). The overall survival analysis evaluated using the median SMD as well as comparisons between the upper 75% and lower 25%, showed a significant difference with *p* < 0.05. In contrast, no significant difference was observed when comparing overall survival using the median SMI and BMI, with *p* > 0.05. Patients with cancer of unknown primary, CUP; 95% Confidence interval, 95% CI.

### 3.4. Univariate and Multivariate Survival Analysis: Identifying Prognostic Factors

Prognostic factors for CUP identified through univariate analysis using a threshold of *p* < 0.1 included age (*p* < 0.001), ECOG-PS (*p* < 0.001), histology (*p* = 0.001), disease extent (*p* < 0.001), SMD (*p* < 0.001), CCI (*p* < 0.001), BMI (*p* = 0.082), and treatment (*p* < 0.001) (Table 3). However, when these factors were evaluated using multivariate analysis, age (*p* = 0.590), ECOG-PS (*p* = 0.061), histology (*p* = 0.100), NLR (*p* = 0.058), CCI (*p* = 0.731), and BMI (*p* = 0.264) were not statistically significant; disease extent, SMD, and treatment remained significant with a *p* < 0.05.

## 4. Discussion

This study analyzed the correlation between CT-based SMD and other clinical factors in patients with CUP and evaluated the prognostic significance of SMD. Our results show that SMD was significantly positively correlated with SMI and negatively correlated with age, NLR, CCI, and ECOG-PS. A trend of decreasing SMD was observed with increasing CCI and ECOG-PS, indicating that a higher number of comorbidities and poorer performance status were associated with a lower SMD. These findings suggest that SMD may serve as an indicator of physical reserve. In multivariate analysis, SMD demonstrated greater statistical significance than ECOG-PS, suggesting that SMD may serve as a more robust prognostic indicator in this cohort.

The Culine Prognostic Model [13] was used to evaluate ECOG-PS, LDH, and liver metastasis in previous prognostic prediction studies. This model has the advantage of being simple to use for prognostic assessment, as it utilizes ECOG-PS, which is widely studied and easily assessed for on-site LDH and liver metastasis. However, the ECOG-PS is confined to a scale of 0–5, which restricts patient evaluation and is subject to the subjectivity of medical personnel. Conversely, the nomogram proposed by Raghav et al. [14], which evaluates sex, ECOG-PS, histology, number of metastatic sites, and NLR, allows for a more comprehensive prediction of 1-year and 2-year outcomes. Despite its strengths, it is still constrained by its dependence on ECOG-PS.

In contrast, this study demonstrated that CT-based SMD allows for a simple and reproducible evaluation of individual patient conditions using a scale ranging from 13 to 60, providing an objective measure that is consistent. SMD was also correlated with other prognostic factors, including ECOG-PS, and multivariate analysis of survival confirmed that SMD was more statistically significant than ECOG-PS [29].

The reason SMD emerged as a more significant prognostic factor than ECOG-PS could be that, as shown in this study, it closely correlates with age, comorbidities, and ECOG-PS, reflecting these factors more comprehensively. Importantly, these correlations remained consistent across both male and female subgroups. This suggests that SMD may objectively represent performance status and physical reserves more effectively. Considering these points, muscle quality, as measured by SMD, may be a more prognostic and accurate indicator of physical reserve than ECOG-PS.

The prognostic value of muscle indicators measured by CT has been widely reported in the literature. Martin et al. demonstrated that both the SMI and SMD are independent prognostic factors across various cancer types [30], and a meta-analysis by Shachar et al. further confirmed a significant association between SMI and overall survival in multiple malignancies [31]. In addition, SMD has been shown to serve as a significant prognostic marker in patients receiving targeted therapy for metastatic renal cell carcinoma [32] and in those with metastatic breast cancer treated with a CDK4/6 inhibitor combined with endocrine therapy [33]. Several studies have also suggested that SMD may outperform SMI in predicting clinical outcomes. For example, Kim et al. reported that among patients with pancreatic cancer undergoing palliative chemotherapy, SMD demonstrated superior prognostic value compared to SMI [34], and Hayashi et al. presented similar findings in metastatic gastric cancer patients receiving palliative chemotherapy [35].

Our study explored the association between SMD and other established prognostic factors in CUP, a heterogeneous group of malignancies, and demonstrated that SMD is a more reliable prognostic marker than SMI. Notably, SMD exhibited stronger prognostic relevance than the subjective ECOG performance status, underscoring the utility of this objective, imaging-based parameter. These findings contribute to the novelty and clinical significance of our study.

Although the cohort size of this study was insufficient to develop a meaningful prediction model, future studies with larger patient populations should explore and compare the roles of SMD and ECOG-PS in prediction models, alongside other factors such as disease extent and NLR, to determine the optimal model for prognostication.

Nonetheless, this study has several limitations. First, it was a single-center retrospective study. Second, the sample size was relatively small due to the rarity of CUP. Finally, while the ECOG-PS is supported by substantial, well-established data, the SMD has yet to be fully validated as an indicator of physical reserve.

Therefore, future studies should involve larger sample sizes and utilize abdominal CT to assess SMD more precisely, aiming to clarify whether SMD can replace ECOG-PS or serve as a complementary prognostic tool. In addition, multi-center studies are necessary to minimize potential biases inherent in single-center research and to enhance the generalizability of findings. Moreover, it is essential to investigate whether SMD can serve as a prognostic surrogate for ECOG-PS in other malignancies beyond CUP, thereby expanding its potential utility across a broader range of cancer types.

## 5. Conclusions

SMD, as a measure of muscle quality, demonstrates superior prognostic value compared to the subjective ECOG-PS and may serve as a reliable objective tool for assessing physical reserve in patients with CUP.

## Figures and Tables

**Table 1:** Baseline demographic and clinical characteristics of the patients.

Variables	Number (Total *n* = 184, %)
Age (years)	
<65	72 (39.1%)
≥65	112 (60.9%)
Sex	
Female	82 (44.6%)
Male	102 (55.4%)
Histology	
Adenocarcinoma	58 (31.5%)
Squamous cell carcinoma	35 (19.0%)
Poorly differentiated carcinoma	33 (17.9%)
Neuroendocrine cell carcinoma	16 (8.7%)
Small cell carcinoma	4 (2.2%)
Undifferentiated carcinoma	3 (1.6%)
Others	35 (19.0%)
ECOG-PS	
0–2	157 (85.3%)
3–4	27 (14.7%)
CCI (Charlson Comorbidity Index) *	
≤3	119 (64.7%)
>3	65 (35.3%)
NLR (Neutrophil-to-lymphocyte ratio)	
<5	100 (54.3%)
≥5	84 (45.7%)
Disease extent	
Single or oligometastasis	79 (42.9%)
Multiple	105 (57.1%)
SMD (Skeletal Muscle Density)	
≤39	69 (37.5%)
>39	63 (34.2%)
Not available	52 (28.3%)
SMI (Skeletal Muscle Mass Index)	
≤43.3	63 (34.2%)
>43.3	62 (33.7%)
Not available	59 (32.1%)
BMI (Body Mass Index)	
≤23.05	62 (33.7%)
>23.05	63 (34.2%)
Not available	59 (32.1%)
Treatment	
Chemotherapy group	60 (32.6%)
Local treatment group	48 (26.1%)
No (supportive care group)	76 (41.3%)

* The CCI includes factors such as age, myocardial infarction, congestive heart failure, peripheral vascular disease, cerebrovascular accident, dementia, chronic obstructive pulmonary disease, connective tissue disease, peptic ulcer disease, liver disease, diabetes mellitus, hemiplegia, moderate-to-severe chronic kidney disease, solid tumor, leukemia, lymphoma, and acquired immune deficiency syndrome. The CCI scores range from 0 to 37 points [26,28].

**Table 2:** Pearson and Spearman correlation between clinical factors of the study patients.

Correlation		All (*n* = 132)		Male (*n* = 74)		Female (*n* = 58)	
		r	*p* Value	r	*p* Value	r	*p* Value
Pearson	SMD vs. Age	−0.510	<0.001	−0.511	<0.001	−0.542	<0.001
	SMD vs. NLR	−0.205	0.019	−0.262	0.024	−0.134	0.317
	SMD vs. SMI	0.341	<0.001	0.418	<0.001	−0.006	0.967
	SMD vs. BMI	−0.145	0.114	−0.039	0.750	−0.174	0.217
	SMI vs. Age	−0.343	<0.001	−0.441	<0.001	−0.177	0.204
	SMI vs. NLR	−0.273	0.002	−0.329	0.005	−0.214	0.124
	SMI vs. BMI	0.453	<0.001	0.609	<0.001	0.497	<0.001
	BMI vs. Age	−0.228	0.011	−0.373	0.001	−0.078	0.578
	BMI vs. NLR	−0.246	0.006	−0.358	0.002	−0.160	0.254
Spearman	SMD vs. CCI	−0.443	<0.001	−0.476	<0.001	−0.457	<0.001
	SMD vs. ECOG-PS	−0.488	<0.001	−0.523	<0.001	−0.486	<0.001
	SMI vs. CCI	−0.309	0.001	−0.441	<0.001	−0.109	0.435
	SMI vs. ECOG-PS	−0.224	0.013	−0.349	0.003	−0.107	0.446
	BMI vs. CCI	−0.143	0.113	−0.228	0.054	−0.003	0.984
	BMI vs. ECOG-PS	−0.131	0.147	−0.259	0.028	0.052	0.710

SMD was significantly associated with age, CCI, and ECOG-PS, regardless of sex. In contrast, SMI showed significant correlations with CCI and ECOG-PS only in the male subgroup, with no significant associations observed in females. SMD, skeletal muscle density; NLR, neutrophil-to-lymphocyte ratio; SMI, skeletal muscle index; BMI, body mass index; CCI, Charlson Comorbidity Index; ECOG-PS, Eastern Cooperative Oncology Group Performance Status.

**Table 3:** Univariate and multivariate analyses of prognostic factors of the study patients.

Variable	Univariate		Multivariate	
	HR (95% CI)	*p* Value	HR (95% CI)	*p* Value
Sex (male)	1.209 (0.880–1.659)	0.241		
Age *	1.030 (1.016–1.044)	<0.001	0.992 (0.964–1.021)	0.590
ECOG-PS (0–4)	1.935 (1.658–2.258)	<0.001	1.275 (0.989–1.644)	0.061
Histology	1.775 (1.271–2.478)	0.001	1.498 (0.925–2.427)	0.100
(adenocarcinoma)				
Disease extent	3.800 (2.686–5.375)	<0.001	2.254 (1.331–3.819)	0.003
(multiple)				
NLR *	1.051 (1.024–1.078)	<0.001	1.041 (0.999–1.085)	0.058
SMD *	0.954 (0.934–0.975)	<0.001	0.962 (0.937–0.987)	0.004
CCI (0~8)	1.222 (1.113–1.341)	<0.001	0.965 (0.790–1.180)	0.731
SMI *	0.987 (0.965–1.011)	0.283		
BMI *	0.950 (0.897–1.006)	0.082	0.966 (0.909–1.026)	0.264
Treatment (no treatment)	1 (reference)		1 (reference)	
Local treatment	0.126 (0.069–0.232)	<0.001	0.230 (0.100–0.528)	<0.001
Chemotherapy	0.195 (0.136–0.279)	<0.001	0.357 (0.177–0.720)	0.004

HR, Hazard Ratio; CI, Confidence Interval; * indicates continuous variables. ECOG-PS, Eastern Cooperative Oncology Group Performance Status; NLR, Neutrophil-to-lymphocyte ratio; SMD, Skeletal Muscle Density; CCI, Charlson Comorbidity Index; SMI, Skeletal Muscle Index; BMI, Body Mass Index; HR, Hazard Ratio; CI, Confidence Interval.

## Data Availability

The datasets used and/or analyzed during the current study are available from the corresponding author on reasonable request.

## Supplementary Materials

The following supporting information can be downloaded at https://www.mdpi.com/article/10.3390/jcm14092947/s1; Figure S1: The schematic diagram of the deep learning-based muscle analysis method (ClariMetabo); Figure S2: Example of a muscle analysis report; Table S1: Site of involvement at diagnosis in CUP patients; Table S2: Initial treatment after diagnosis; Table S3: 1st line chemotherapy regimen as the first treatment after diagnosis; Table S4: 2nd line chemotherapy regimen; Table S5: Subgroup Analysis by Sex.

## Abbreviations

The following abbreviations are used in this manuscript:

Cancer of Unknown Primary origin	CUP
Eastern Cooperative Oncology Group-Performance Status	ECOG-PS
Lactate dehydrogenase	LDH
Neutrophil-to-lymphocyte ratio	NLR
Computed tomography	CT
Skeletal muscle index	SMI
Skeletal muscle density	SMD
Hounsfield units	HU
Overall survival	OS
Charlson Comorbidity Index	CCI
Median overall survival	mOS
