# Supplementary material for: Skeletal Muscle Density as a Predictor of Prognosis and Physical Reserve in Patients with Cancer of Unknown Primary
Source: J Clin Med. 2025 Apr 24;14(9):2947. doi: 10.3390/jcm14092947 (PMC12072687; doi:10.3390/jcm14092947)
Supplement: Supplementary file 1 [file jcm-14-02947-s001.zip › Supplementary Table S1. Site of involvement at diagnosis in CUP patients..docx]

**Supplementary Table S1. Site of involvement at diagnosis in CUP patients.**

| **Site of involvement** | **Number (total n=184, %)** |
| --- | --- |
| Lymph node | 134(72.8%) |
| Bone | 63(34.2%) |
| Lung | 40(21.7%) |
| Liver | 37(20.1%) |
| Pleura/Pleural effusion | 36(19.6%) |
| Omentum/Peritoneum/Ascites/Mesentery | 33(17.9%) |
| Soft tissue | 16(8.7%) |
| Adrenal gland | 16(8.7%) |
| Kidney/Ureter/Bladder | 8(4.3%) |
| Brain | 7(3.8%) |
| Prostate/Testis/Scrotum/Uterus/Ovary | 7(3.8%) |
| Stomach/Small intestine/Large intestine/Rectum/Anus | 7(3.8%) |
| Pericardium/Pericardial effusion | 7(3.8%) |
| Spleen | 3(1.6%) |
| Pancreas | 3(1.6%) |
| Thyroid | 3(1.6%) |
| Head & neck | 2(1.1%) |
| Other seeding meta | 2(1.1%) |
| Thymus | 1(0.5%) |
| Skin | 1(0.5%) |
| Gall bladder/Common bile duct | 0(0.0%) |
| Breast | 0(0.0%) |
