# Supplementary material for: Skeletal Muscle Density as a Predictor of Prognosis and Physical Reserve in Patients with Cancer of Unknown Primary
Source: J Clin Med. 2025 Apr 24;14(9):2947. doi: 10.3390/jcm14092947 (PMC12072687; doi:10.3390/jcm14092947)
Supplement: Supplementary file 1 [file jcm-14-02947-s001.zip › Supplementary Table S2. Initial treatment after diagnosis..docx]

**Supplementary Table S2. Initial treatment after diagnosis.**

| **Treatments** | **Number (total n=184, %)** |
| --- | --- |
| Chemotherapy | 60(32.6%) |
| Radiotherapy | 13(7.1%) |
| curative | 3(1.6%) |
| palliative | 10(5.4%) |
| Concurrent chemo-radiation therapy after operation | 11(6.0%) |
| Concurrent chemo-radiation therapy | 9(4.9%) |
| Operation with adjuvant radiotherapy | 8(4.3%) |
| Chemotherapy with radiotherapy | 3(1.6%) |
| Operation | 2(1.1%) |
| Operation with adjuvant chemotherapy | 1(0.5%) |
| Operation with adjuvant chemotherapy and radiotherapy | 1(0.5%) |
| **No treatment** | 76(41.3%) |
