# Supplementary material for: Skeletal Muscle Density as a Predictor of Prognosis and Physical Reserve in Patients with Cancer of Unknown Primary
Source: J Clin Med. 2025 Apr 24;14(9):2947. doi: 10.3390/jcm14092947 (PMC12072687; doi:10.3390/jcm14092947)
Supplement: Supplementary file 1 [file jcm-14-02947-s001.zip › Supplementary Table S3. 1st line chemotherapy regimen as the first treatment after diagnosis.docx]

**Supplementary Table S3. 1^st^ line chemotherapy regimen as the first treatment after diagnosis**

| **1^st^ line chemotherapy regimen** | **Number (total n=60, %)** |
| --- | --- |
| PC (Paclitaxel/Carboplatin) | 25(41.7%) |
| EP (Etoposide/Cisplatin) | 10(16.7%) |
| FP (5-Fluorouracil/Cisplatin) | 6(10.0%) |
| GP (Gemcitabine/Cisplatin) | 4(6.7%) |
| ICE (Ifosfamide/Carboplatin/Etoposide) | 4(6.7%) |
| DCF (Docetaxel/Cisplatin/5-Fluorouracil) | 2(3.3%) |
| ECF (Epirubicin/Cisplatin/5-Fluorouracil) | 2(3.3%) |
| GC (Gemcitabine/Carboplatin) | 1(1.7%) |
| Gemcitabine | 1(1.7%) |
| Others | 5(8.3%) |
