# Supplementary material for: Skeletal Muscle Density as a Predictor of Prognosis and Physical Reserve in Patients with Cancer of Unknown Primary
Source: J Clin Med. 2025 Apr 24;14(9):2947. doi: 10.3390/jcm14092947 (PMC12072687; doi:10.3390/jcm14092947)
Supplement: Supplementary file 1 [file jcm-14-02947-s001.zip › Supplementary Table S4. 2nd line chemotherapy regimen..docx]

**Supplementary Table S4. 2^nd^ line chemotherapy regimen.**

| **2^nd^ line chemotherapy regimen** | **Number (total n=33, %)** |
| --- | --- |
| GP (Gemcitabine/Cisplatin) | 10(30.3%) |
| EP (Etoposide/Cisplatin) | 3(9.1%) |
| PC (Paclitaxel/Carboplatin) | 3(9.1%) |
| CAV (Cyclophosphamide/Doxorubicin/Vincristine) | 2(6.1%) |
| Docetaxel | 2(6.1%) |
| IP(Irinotecan/Cisplatin) | 2(6.1%) |
| FP (5-Fluorouracil/Cisplatin) | 1(3.0%) |
| GP (Gemcitabine/Cisplatin) | 1(3.0%) |
| Others | 9(27%) |
