# Supplementary material for: Skeletal Muscle Density as a Predictor of Prognosis and Physical Reserve in Patients with Cancer of Unknown Primary
Source: J Clin Med. 2025 Apr 24;14(9):2947. doi: 10.3390/jcm14092947 (PMC12072687; doi:10.3390/jcm14092947)
Supplement: Supplementary file 1 [file jcm-14-02947-s001.zip › Supplementary Table S5. Subgroup analysis-sex.docx]

**Supplementary Table S5. Subgroup Analysis by Sex**

| **Variable** | **Univariate** | |
| --- | --- | --- |
|  | **HR (95% CI)** | ***P* value** |
| Age* | 1.035 (1.012–1.059) | 0.003 |
| ECOG-PS (0–4) | 2.020 (1.586–2.572) | < 0.001 |
| Histology | 2.020 (1.179–3.46) | 0.011 |
| (adenocarcinoma) |  |  |
| Disease extent | 4.757 (2.769-8.173) | < 0.001 |
| (multiple) |  |  |
| NLR* | 1.036 (0.998-1.077) | 0.065 |
| SMD* | 0.929 (0.897–0.963) | < 0.001 |
| CCI (0~8) | 1.241 (1.060–1.453) | 0.007 |
| SMI* | 1.022 (0.978–1.068) | 0.325 |
| BMI* | 1.027 (0.950–1.111) | 0.501 |
| Treatment  Local treatment  Chemotherapy | 0.058 (0.025-0.138)  0.184 (0.102-0.335) | < 0.001  < 0.001 |

**<Male> <Female>**

| **Variable** | **Univariate** | |
| --- | --- | --- |
|  | **HR (95% CI)** | ***P* value** |
| Age* | 1.028 (1.011–1.045) | 0.001 |
| ECOG-PS (0–4) | 1.830 (1.495–2.240) | < 0.001 |
| Histology | 1.558 (1.011–2.400) | 0.045 |
| (adenocarcinoma) |  |  |
| Disease extent | 3.084 (1.958–4.857) | < 0.001 |
| (multiple) |  |  |
| NLR* | 1.078 (1.037–1.120) | < 0.001 |
| SMD* | 0.949 (0.920–0.979) | < 0.001 |
| CCI (0~8) | 1.200 (1.070–1.346) | 0.002 |
| SMI* | 0.934 (0.899–0.970) | <0.001 |
| BMI* | 0.896 (0.826–0.972) | 0.008 |
| Treatment  Local treatment  Chemotherapy | 0.174 (0.097-0.313)  0.303 (0.184-0.498) | < 0.001  < 0.001 |

**HR, Hazard Ratio; CI, Confidence Interval; *indicates continuous variables.**

ECOG-PS, Eastern Cooperative Oncology Group Performance Status ; NLR, Neutrophil-to-lymphocyte ratio; SMD, Skeletal Muscle Density; CCI, Charlson Comorbidity Index; SMI, Skeletal Muscle Index; BMI, Body Mass Index; HR, Hazard Ratio; CI, Confidence Interval.
