# Supplementary figures and images for: Skeletal Muscle Density as a Predictor of Prognosis and Physical Reserve in Patients with Cancer of Unknown Primary
Source: J Clin Med. 2025 Apr 24;14(9):2947. doi: 10.3390/jcm14092947 (PMC12072687; doi:10.3390/jcm14092947)

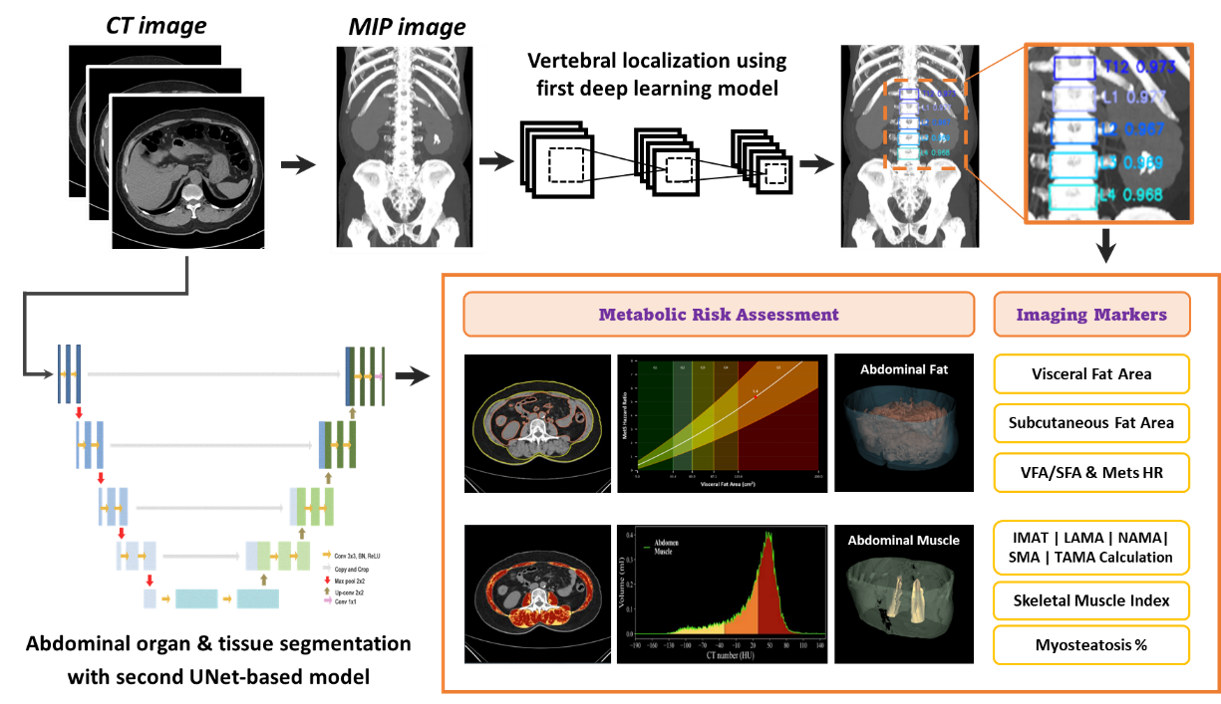

Supplement: Supplementary file 1 [file jcm-14-02947-s001.zip › Figure S1.tif]

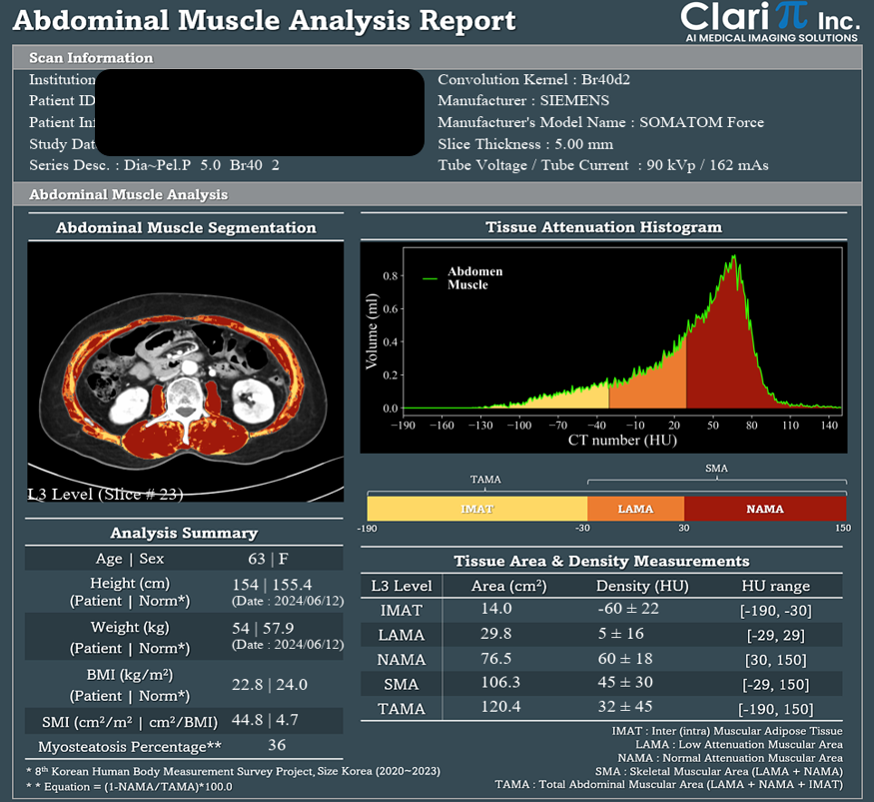

Supplement: Supplementary file 1 [file jcm-14-02947-s001.zip › Figure S2.png]
